# Transcallosal and Corticospinal White Matter Disease and Its Association With Motor Impairment in Multiple Sclerosis

**DOI:** 10.3389/fneur.2022.811315

**Published:** 2022-06-15

**Authors:** Keejin Yoon, Derek B. Archer, Margareta A. Clarke, Seth A. Smith, Ipek Oguz, Gary Cutter, Junzhong Xu, Francesca Bagnato

**Affiliations:** ^1^Neuroimaging Unit, Division of Neuroimmunology, Department of Neurology, Vanderbilt University Medical Center, Nashville, TN, United States; ^2^College of Arts and Sciences, Vanderbilt University, Nashville, TN, United States; ^3^Vanderbilt Memory and Alzheimer's Center, Vanderbilt University School of Medicine, Nashville, TN, United States; ^4^Vanderbilt University School of Medicine, Vanderbilt Genetics Institute, Nashville, TN, United States; ^5^Department of Radiology and Radiological Sciences, Vanderbilt University Institute of Imaging Science, Vanderbilt University Medical Center, Nashville, TN, United States; ^6^Department of Science, Vanderbilt University, Nashville, TN, United States; ^7^Department of Biostatistics, School of Public Health, The University of Alabama at Birmingham, Birmingham, AL, United States; ^8^Department of Neurology, VA Medical Center, TN Valley Healthcare System, Nashville, TN, United States

**Keywords:** multiple sclerosis, magnetic resonance imaging (MRI), neurite orientation dispersion and density imagining (NODDI), neurodegeneration, probabilistic tractography

## Abstract

**Purpose:**

In this cross-sectional, proof-of-concept study, we propose that using the more pathologically-specific neurite orientation dispersion and density imaging (NODDI) method, in conjunction with high-resolution probabilistic tractography, white matter tract templates can improve the assessment of regional axonal injury and its association with disability of people with multiple sclerosis (pwMS).

**Methods:**

Parametric maps of the neurite density index, orientation dispersion index, and the apparent isotropic volume fraction (IVF) were estimated in 18 pwMS and nine matched healthy controls (HCs). Tract-specific values were measured in transcallosal (TC) fibers from the paracentral lobules and TC and corticospinal fibers from the ventral and dorsal premotor areas, presupplementary and supplementary motor areas, and primary motor cortex. The nonparametric Mann–Whitney *U* test assessed group differences in the NODDI-derived metrics; the Spearman's rank correlation analyses measured associations between the NODDI metrics and other clinical or radiological variables.

**Results:**

IVF values of the TC fiber bundles from the paracentral, presupplementary, and supplementary motor areas were both higher in pwMS than in HCs (*p* ≤ 0.045) and in pwMS with motor disability compared to those without motor disability (*p* ≤ 0.049). IVF in several TC tracts was associated with the Expanded Disability Status Scale score (*p* ≤ 0.047), while regional and overall lesion burden correlated with the Timed 25-Foot Walking Test (*p* ≤ 0.049).

**Conclusion:**

IVF alterations are present in pwMS even when the other NODDI metrics are still mostly preserved. Changes in IVF are biologically non-specific and may not necessarily drive irreversible functional loss. However, by possibly preceding downstream pathologies that are strongly associated with disability accretion, IVF changes are indicators of, otherwise, occult prelesional tissue injury.

## Introduction

Axonal damage is an integral component of multiple sclerosis (MS) pathology and a major cause of neurological impairment in patients (1). Yet, currently available clinical magnetic resonance imaging (MRI) techniques lack the ability to identify and quantify axonal injury *in vivo* (2, 3). More sensitive neuroimaging biomarkers portending disability accretion in person with MS (pwMS) are needed. Those biometrics would allow a more granular understanding of individual likelihood of clinical deterioration as well as identifying windows for earlier treatment interventions.

Advanced multi-b-shell diffusion-based MRI has been proposed as a method to indirectly infer on axonal integrity *in vivo*, with a higher degree of pathological accuracy. The neurite orientation dispersion and density imaging (NODDI) model (4) is one of these newly developed diffusion methods. Compared to the traditional diffusion tensor imaging (DTI) model, the NODDI adds an extra layer of pathological specificity to tissue injury. This is achieved by distinguishing signals from the intracellular (intra-axonal or intraneurite) and extracellular water compartments via three practical metrics, i.e., the neurite density index (NDI), the isotropic volume fraction (IVF), and the orientation dispersion index (ODI) (4, 5). The NDI estimates the volume fraction that is occupied by neurites (axons and dendrites); the IVF represents the volume fraction of water characterized by isotropic diffusion, which in human brains can represent the voxel volume fraction of extracellular fluid; the ODI characterizes neurite orientation and alignment in white matter (WM) fibers, which may lose complexity within advanced pathology (4, 5). Previous studies from our group and that of others demonstrated the sensitivity of the NODDI metrics to tissue injury in MS. However, NODDI-clinical correlations have found to be inconsistent thus far (6). We have also shown that the NODDI-derived metrics do not improve these correlations when compared with the DTI-derived axial diffusivity (AD) (7).

Tractography allows for *in-vivo* delineation of WM tract architecture, enhancing regional specificity. (8, 9). A few studies have been performed combining DTI with tractography of corticospinal (CS) tracts to assess association between disease localized in this tract and disability in pwMS. The CS tract has been the focus of these earlier studies because it is one of the largest tracts of the brain, yielding to its accurate identification with tractography. Furthermore, motor disability is a key component of clinical decline in MS and it is reliably tested in clinical practice (10–16). Changes in fractional anisotropy of the pyramidal tract explained part of the variance of the motor score (12) of the Expanded Disability Status Scale (EDSS) (17), as well as the overall EDSS score (12, 14–16) in previous studies. However, these results were not consistent across studies as some authors found that only the radial diffusivity (11, 16) of the pyramidal tract correlated with the EDSS score or the Timed 25-Foot Walking (T25-FW) Test (18).

Here, we build on previous knowledge to expand the assessment of the relationships between measures of disability and those of regional disease. We reasoned that coupling high-resolution WM tractography analysis with the NODDI would allow exploring MRI-clinical associations in a more pathologically-, but also topographically-specific manner, thus potentially enhancing MRI-clinical correlative analyses. We then confirmed the validity of our data using biometrics derived from two additional microstructural models, e.g., AD from DTI and the apparent axonal volume fraction (V_ax_) from the spherical mean technique (SMT) (19).

In a novel fashion relative to previous literature, in this proof-of-concept study, we used WM tractography templates created from the high-resolution images of the Human Connectome Project database (HCP) (9, 20–22) to obtain an accurate identification of sensorimotor CS tracts descending from motor and premotor areas in addition to the homologous transcallosal (TC) tracts projecting between motor and higher motor areas. As these tracts all contribute to different aspects of human motor skills, we expect our investigations to provide complementary and novel insights on the regional determinant of physical impairment of pwMS.

## Materials and Methods

### Study Design and Cohort

This study was approved by the Vanderbilt University Medical Center Institutional Review Board and was performed in accordance with the Declaration of Helsinki criteria. A signed consent form was obtained from each subject prior to all the examinations. Eighteen pwMS (23) and nine age- and sex-matched healthy controls (HCs) were enrolled over a time span of 6 months. None of the subjects had any contraindications to study MRI or any vascular, immunological, neurodegenerative, and infectious comorbidities that could bias the results. pwMS were at least 6 months free from a clinical relapse, steroid treatment, and changes in disability. All the subjects had a clinical postcontrast brain and spinal cord MRI within 6 months of this initial study, showing no active lesions. Each pwMS underwent a clinical assessment using the EDSS (17) and the T25-FW (18) scores within 2 weeks of the study MRI acquisition. Table 1 depicts demographic and clinical features of the study cohort.

**Table 1:** Demographic, clinical and imaging features of the study cohort.

	**Persons with multiple sclerosis (n = 18)**	**Healthy controls (n = 9)**
*Age (years)*	45.5 ± 14.6	41.7 ± 10
*Sex (females/males)*	12/6	5/4
*Race*	2 African Americans 16 White	8 White 1 Asian
*MS phenotype (CIS/RRMS/SPMS)*	4/11/3	
*Years of disease*	11.5 ± 10.6	
*EDSS score*	1 (0–6.5)	
*T25-FW (seconds)*	10.37 ± 18.12	
*T1-lesion load (cm^3^)*	7.35 ± 7.46	
*T2-lesion load (cm^3^)*	14.89 ± 21.30	
*BPF*	0.81 ± 0.03	

*Numeric data are expressed in mean ± standard deviation except for the EDSS which is reported as median (minimum-maximum value). BPF, brain parenchyma fraction; CIS, clinically isolated syndrome; cm^3^, cubic centimeters; EDSS, expanded disability status scale; RRMS, relapsing remitting multiple sclerosis; SPMS, secondary progression multiple sclerosis; T25-FW, timed 25-foot walk*.

### MRI Acquisition Protocol and Parametric Maps Reconstruction

Scans were acquired using a whole-body 3.0 Tesla (3T) dStream MRI Scanner (Philips Healthcare, Best, The Netherlands) equipped with a volume transmit 32-channel receiver head coil (Nova Medical, Wilmington, Massachusetts, USA). The scanning protocol included: T1-weighted (T1W) fast field echo and T2-weighted (T2W) turbo spin echo (TSE), T2W fluid-attenuated inversion recovery (FLAIR) sequences, and a multi-b shells diffusion-weighted imaging sequence, the parameters of which are presented in Table 2.

**Table 2:** Multi-slice multi-shell diffusion-weighted imaging parameters.

**T1W FFE / T2W TSE / T2W FLAIR**		**SMT (single-shot, EPI)**
TE	4.6 / 80 / 125 ms	74 ms
TR	508 / 4,000 / 11,000 ms	13.5 seconds
Resolution	0.4x0.4x2 mm^3^	2x2x2 mm^3^
Number of b-shells	N/A	2 (1000, 2500 s/mm^3^)
Diffusion directions	N/A	90 (45 on each b shell)

*B, diffusion weighting factor; EPI, echo planar imaging; FFE, fast field echo; FLAIR, fluid-attenuated inversion recovery; ms, milliseconds; T1W, T1-weighted; T2W, T2-weighted; TE, echo time; TR, repetition time; TSE, turbo spin echo*.

All the diffusion-weighted images were corrected using FMRIB Software Library (FSL) topup and eddy toolbox (https://fsl.fmrib.ox.ac.uk/fsl/) to remove susceptibility and eddy current-induced distortions. After that, the neurite orientation dispersion and density imaging (NODDI) toolbox (http://mig.cs.ucl.ac.uk/index.php?n=Tutorial.NODDImatlab) was used to reconstruct all the NODDI parametric maps (24–26). AD and V_ax_ were fitted as previously reported (27).

Lastly, each map was co-registered and resampled to the T2W TSE image as an affine co-registration using the FMRIB's Linear Image Registration Tool (FLIRT) toolbox in FSL (https://fsl.fmrib.ox.ac.uk/fsl/).

### Overall Lesion Burden and Brain Atrophy Calculation

Overall lesion load was calculated by manually segmenting chronic T2 lesions (28) and chronic black holes (29) on T2W FLAIR and T1W fast field echo, respectively, using graphic tools in Medical Image Processing, Analysis, and Visualization (MIPAV) (http://mipav.cit.nih.gov/), in accordance with lesion definitions previously established. Afterward, and we used MATrix LABoratory (MATLAB) (Mathworks, Natick, Massachusetts, USA, version R2019A) to subtract the chronic black hole mask from that of T2 lesions. This calculation was done to avoid accounting for chronic black hole volume twice. Brain atrophy was measured using the brain parenchymal fraction (BPF), calculated as the ratio of the parenchymal volume to the intracranial volume and derived using an automatic tissue segmentation method with publicly available software and atlases (30–33).

### Calculation of the Tract-Specific AD, Vax and NODDI-Derived Metrics

**Figure 1:**
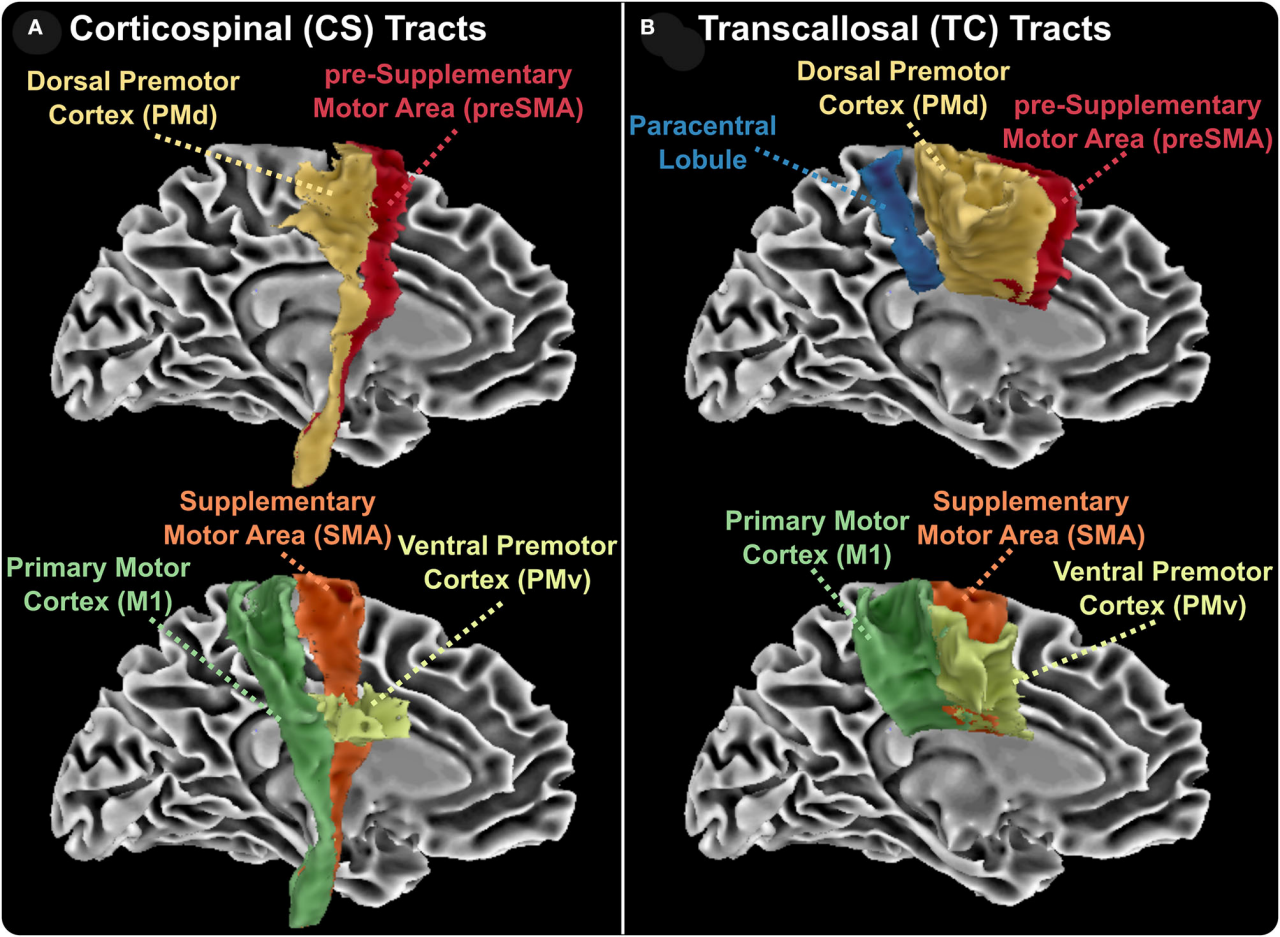
Reconstructed white matter tracts. Corticospinal (CS, **A**) and transcallosal (TC, **B**) aspects of reconstructed white matter tracts. CS and TC tracts include the ventral premotor cortex, the dorsal premotor cortex, the presupplementary area, the supplementary motor area, and primary motor cortex. TC tracts also include the interhemispheric bundles connecting the two paracentral lobes.

To obtain a standardized space registration of all parametric maps, the IVF parametric map was first co-registered to the anatomical T1W TSE image using the Advanced Normalization Tools package (34). The resulting affine matrix and non-linear warp were then applied to the remaining diffusion images. Afterward, a non-linear transformation was used to transform each individual anatomical image to the MNI152 template. The affine matrix and non-linear warp were then applied to all the diffusion images, as well as lesion masks. Well-established tractography templates were collated from several atlases (20–22, 35, 36) and each metric was then calculated within all the tracts using FSL software (version 6.0.1) (37, 38). To assess the effects of anatomically-specific axonal injury on motor disability, we chose WM tracts associated with motor functions. As shown in Figure 1, these tracts included the TC fiber bundles of the paracentral lobules and both the TC and CS fiber bundles of the ventral premotor area, the dorsal premotor area, the supplementary motor area, the presupplementary motor area, and the primary motor cortex.

**Figure 2:**
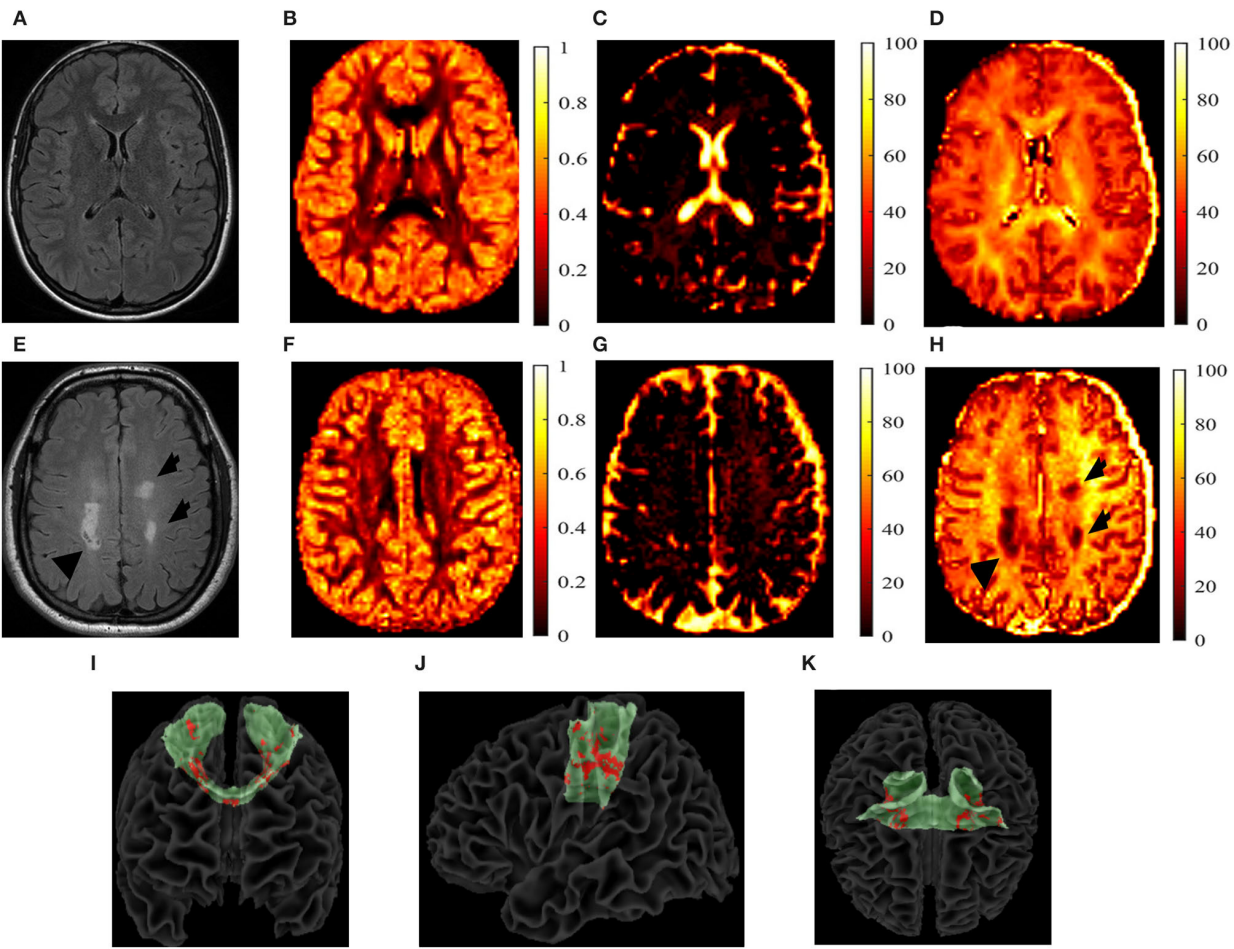
T2-fluid-attenuated inversion recovery (FLAIR) images and the neurite orientation dispersion and density imaging (NODDI) parametric maps. T2-fluid-attenuated inversion recovery (T2-FLAIR) images and the neurite orientation dispersion and density index (NODDI) parametric maps of a 27-year-old, white, female healthy control (HC) **(A–D)** and a 40-year-old white person with relapsing-remitting multiple sclerosis **(E–H)**. The T2-FLAIR **(A,E)**, orientation dispersion index (ODI) **(B,F)**, isotropic volume fraction (IVF) **(C,G)**, and the neurite density index (NDI) **(D,H)** are shown. T2 lesions are indicated by black arrows. Reconstructed transcallosal fibers of the primary motor cortex are shown in green with both the T1 and T2 lesions shown in red with a coronal **(I)**, sagittal **(J)**, and axial **(K)** views. I-K show the coronal **(I)**, sagittal **(J)**, and axial **(K)** views of the reconstructed TC fibers of primary cortex in green, with lesions marked in red of a 56-year-old white male with secondary-progressive multiple sclerosis.

For the pwMS cohort, additional analyses were conducted. First, the chronic black hole lesion masks were used to quantify lesion volumes within each tract. An identical procedure was used for the T2-lesion masks. These masks were subsequently overlaid onto the AD, V_ax_, and NODDI maps and were summed to calculate tract-specific total lesion loads. From these lesion masks, tract-specific lesional and normal-appearing white matter (NAWM) AD, V_ax_, IVF, NDI, and ODI measures were calculated and acquired. All of the aforementioned calculations were done using FSL software (version 6.01) (38, 39). Figure 2 shows example of each parametric map in one pwMS and one HC.

### Statistical Analyses

We derived IVF, ODI, and NDI measurements of both the whole tracts, i.e., inclusive of NAWM and lesions (both T2 lesions and chronic black holes) and NAWM only. For each metric, we first analyzed differences between pwMS (*n* = 18) and HCs (*n* = 9). Then, we assessed differences between pwMS with the EDSS motor score of ≥ 1 (*n* = 6) and those with the normal motor EDSS of 0 (*n* = 12). A cutoff of “1” at the motor score was chosen, as this reflects the minimal degree of impairment that one can detect with the EDSS score and classifies the pwMS group into those with a anormal examination (EDSS = 0) vs. those without (EDSS ≥1).

Percent differences in AD, V_ax_, IVF, ODI, and NDI measurements between pwMS and HCs were also calculated using the formula below, with ***a*** representing tract-specific values averaged across HCs and ***b*** representing tract-specific values averaged across pwMS.

The same formula was used to calculate percent differences between pwMS without (***a***) and with (***b***) motor disability. We chose to calculate percent differences instead of the actual difference to account for the differences in the magnitudes of each metric we analyzed.


$$\begin{array}{l} Percent\;difference=\frac{\left|a-b\right|}{(a+b)/2}\times 100\% \end{array}$$

Group differences in quantitative were compared using the Mann–Whitney *U* test.

The Spearman's rank correlation was used as a non-parametric test to assess associations between pwMS tract metrics and clinical, radiological, and demographic measures.

The Spearman's rank correlations and the Mann–Whitney *U*-tests were chosen for our analyses over other methods due to our small sample size and the EDSS scores not being normally distributed, justifying a non-parametric analysis.

Due to the pilot and exploratory nature of this study, no correction for multiple comparisons was applied.

All the statistical tests were two-sided and a *p*-value ≤ 0.05 was considered as statistically significant. Statistical analyses were performed using MATLAB (*MATLAB version 2019A*).

## Results

All the pwMS presented with lesions on all the examined tracts. Supplementary Table 1 *(on line)* depicts the regional lesion volume measured on each tract in the entire pwMS cohort. The same data for pwMS and different degrees of motor disability are given in Supplementary Table 1 *(on line)*.

### Differences in AD, Vax and NODDI-Derived Metrics Between pwMS and HCs

**Figure 3:**
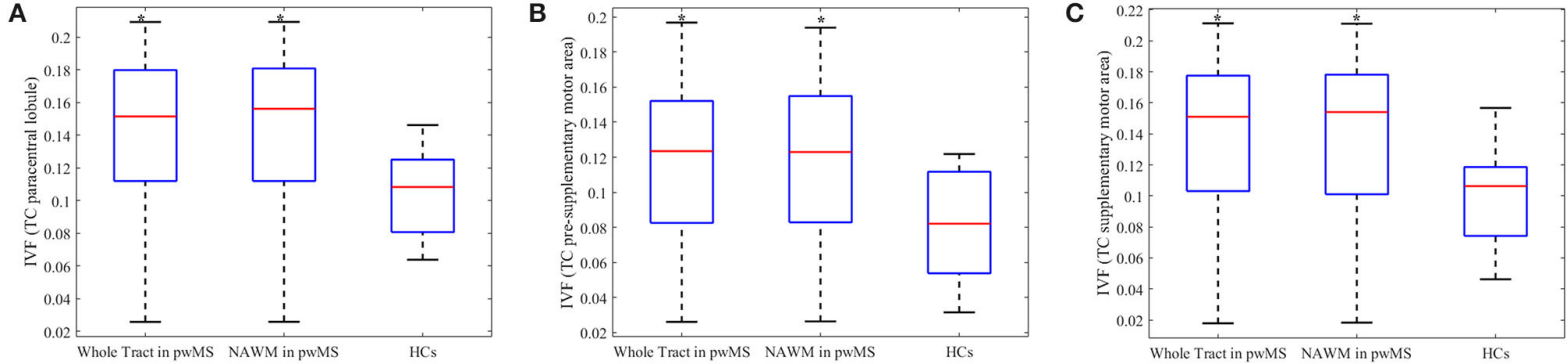
Boxplots of metrics which were significantly different between person with multiple sclerosis and healthy controls. The isotropic volume fraction (IVF) values of the transcallosal (TC) paracentral lobule **(A)**, TC pre-supplementary area **(B)** and TC supplementary motor area tracts **(C)**. The IVF values of whole tract and normal appearing white matter (NAWM) in person with multiple sclerosis (pwMS) and whole tract of healthy controls (HCs) are presented. The upper and lower black horizontal bars represent maximum and minimum values, respectively. The upper and lower blue horizontal bars represent the upper and lower quartiles, while the red bar represents the median of the group. Asterisks indicate the metrics found to be statistically different (*p* ≤ 0.05) in pwMS compared to HCs.

The IVF and AD were the only metrics showing significant group differences. Figure 3 represents the IVF of tracts for which group differences were found to be significant: (1) whole tract (*p* = .018, *U* = 35,000) and NAWM (*p* = 0.018, *U* = 35,000) of the TC fibers connecting the two paracentral lobules, with both percent differences of 29.4% and 29.4%, respectively (Figure 3A); (2) whole tract (*p* = 0.045, *U* = 42,000) and NAWM (*p* = 0.035, *U* = 77,000) of the TC fibers of pre-supplementary motor area with percent differences of 40.7 and 40.5%, respectively (Figure 3B); and (3) whole tract (*p* = 0.045, *U* = 45,000) and NAWM (*p* = 0.035, *U* = 77,000) of the TC fibers of supplementary motor area with percent differences of 34.5 and 34.6%, respectively (Figure 3C). When individuals with secondary progressive MS were removed from the analyses, group differences held true for both the whole tract and NAWM of the TC fibers connecting the two paracentral lobules (*p* = 0.016, *U* = 27,000 for both) and the pre-supplementary motor areas (*p* = 0.040, *U* = 33,000 for both).

AD showed significant group differences for: (1) whole tract (*p* = .018, *U* = 35,000) and NAWM (*p* = .018, *U* = 35,000) of the TC fibers of the pre-supplementary motor area and (2) whole tract (*p* = .029, *U* = 38,500) and NAWM (*p* = .031, *U* = 39,000) of the TC fibers of the supplementary motor area; however, no significant group differences were found in the TC fibers of the two paracentral lobules. No differences were observed in SMT derived Vax on any of the examined tracts.

### Differences in AD, Vax and NODDI-Derived Metrics Between pwMS With and Without Motor Disability

**Figure 4:**
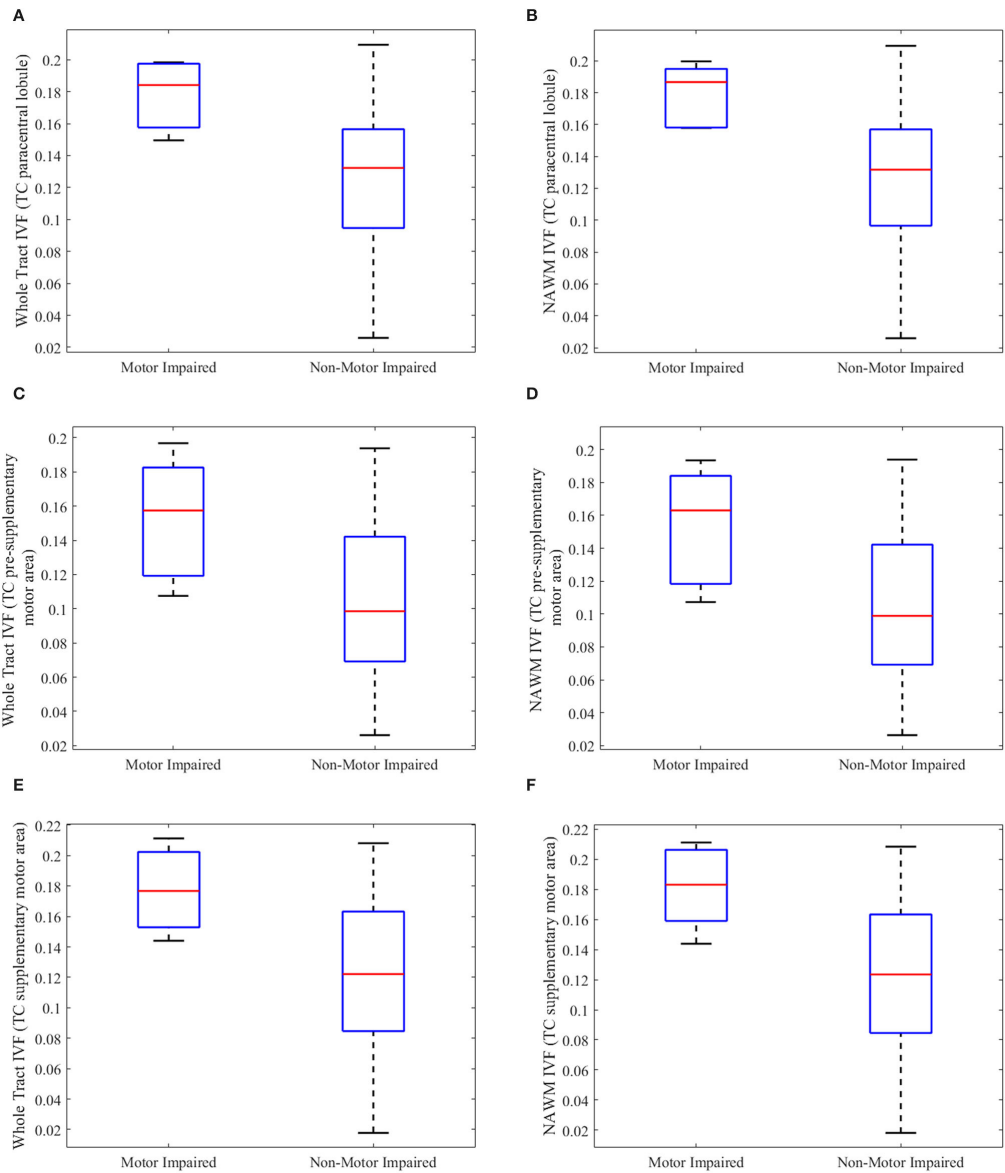
Boxplots of tract metrics which were significantly different between person with multiple sclerosis with and without motor impairment. The isotropic volume fraction (IVF) values measured in the whole tract and normal appearing white matter (NAWM) of the transcallosal (TC) paracentral lobule **(A,B)**, TC presupplementary area **(C,D)**, and TC supplementary motor area tracts **(E,F)** in person with multiple sclerosis (pwMS) and motor impairment and those without motor impairment. **(A,C,E)** show whole-tract IVF values, while **(B,D,F)** show normal appearing white matter (NAWM) IVF values. The upper and lower black horizontal bars represent maximum and minimum values, respectively. The upper and lower blue horizontal bars represent the upper and lower quartiles, while the red bar represents the median of the group.

Subgroup analyses were performed on pwMS with motor disability vs. those without motor disability to further investigate if changes seen in pwMS relative to HCs persisted. pwMS and motor disability were older (*p* = 0.031, *U* = 13,000), had longer disease duration (*p* = 0.003, *U* = 4,000), and higher EDSS scores (*p* = 0.004, *U* = 6,000) than the ones without. As shown in Figure 4, increase in the IVF persisted in the following tracts of motor-impaired pwMS: (1) whole tract (*p* = 0.019, *U* = 11,000) and NAWM (*p* = 0.015, *U* = 10,000) of the TC fibers of the paracentral lobule with percent differences of 18.6 and 20.3%, respectively, in motor impaired vs. non-motor impaired pwMS (Figures 4A,B); (2) whole tract (*p* = 0.049, *U* = 15,000) and NAWM (*p* = 0.039, *U* = 14,000) of the TC fibers of presupplementary motor area with percent differences of 37.5 and 42.4%, respectively (Figures 4C,D); and (3) whole tract (*p* = .039, *U* = 14,000) and NAWM (*p* = 0.031, *U* = 13,000) of the TC fibers of supplementary motor area with percent differences of 29.8 and 33.5%, respectively (Figures 4E,F).

AD showed significant group differences for: (1) whole tract (*p* = 0.013, *U* = 9,500) and NAWM (*p* = 0.022, *U* = 11,500) of the TC fibers of the presupplementary motor area and (2) whole tract (*p* = 0.015, *U* = 10,000) and NAWM (*p* = 0.010, *U* = 8,500) of the TC fibers of the supplementary motor area; however, no significant group differences were found in the TC fibers of the two paracentral lobules. No differences were observed in SMT derived Vax on any of the examined tracts.

### Associations Between AD or NODDI-Derived Metrics and Other MRI/Clinical Measures of Disease

Those analyses focused only on the IVF and AD of the tracts which showed group differences, e.g., pwMS relative to HCs. In those tracts, there were no associations between NAWM, IVF, or AD and regional lesion burden, as shown in Supplementary Table 3 *(online)*.

**Figure 5:**
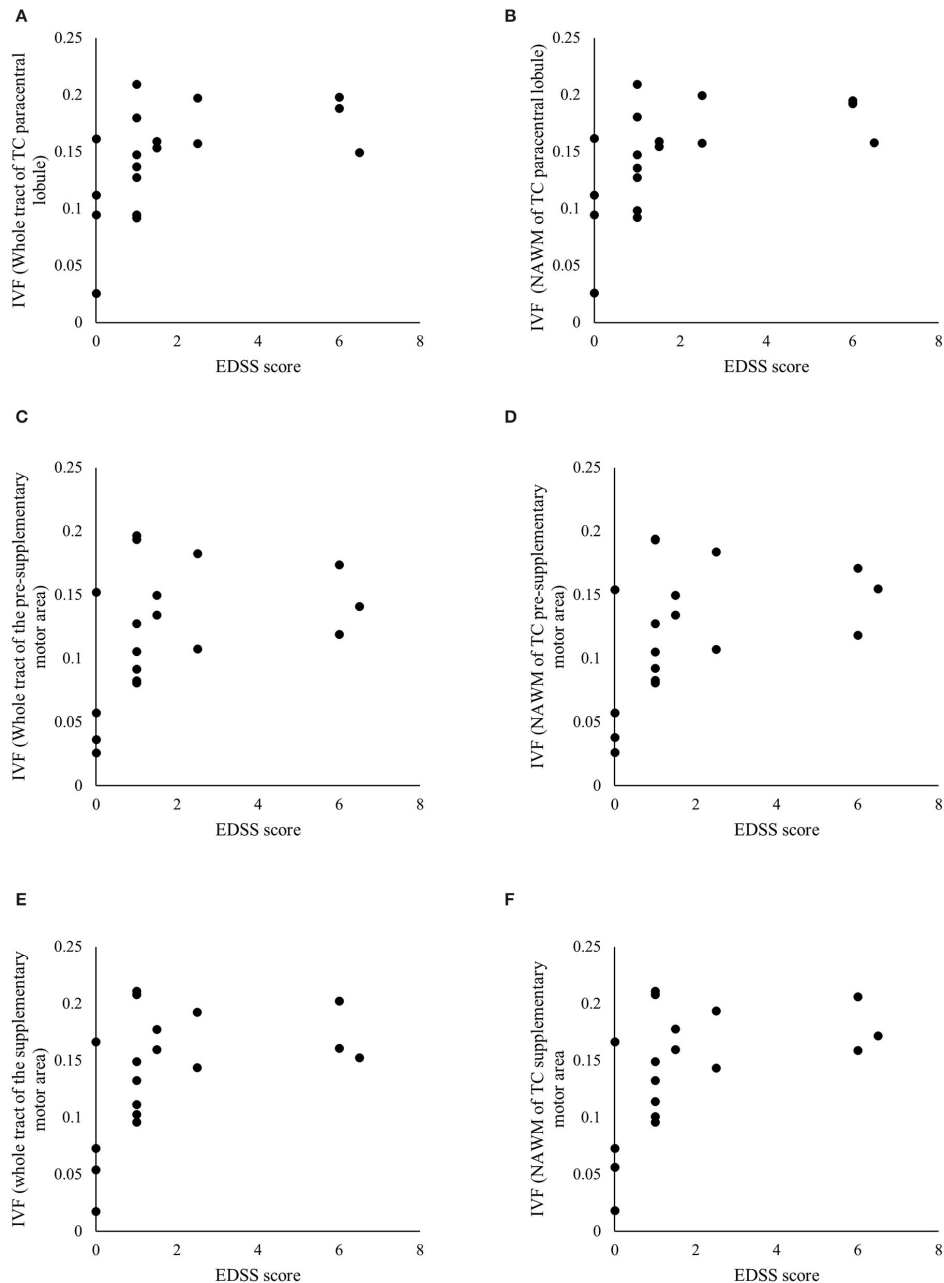
Significant associations between clinical and radiological measures. Scatterplots of the Expanded Disability Status Scale (EDSS) scores and the isotropic volume fraction (IVF) values measured in the whole tract and normal appearing white matter (NAWM) of the transcallosal (TC) paracentral lobule **(A,B)**, TC pre-supplementary motor area **(C,D)**, and TC supplementary motor area **(E,F)**. Table 3 details *p-* and *rho*-values.

The Spearman's correlation analyses yielded several associations between the IVF and AD and disability, as measured by the EDSS score, but not the T25-FW. We depict these significant associations in Table 3 and present the ones for IVF in Figure 5 as well.

**Table 3:** Significant correlations between IVF / AD and EDSS score.

	**Whole tract**	**NAWM**
	**IVF**	
TC paracentral	0.543 (0.020)	0.570 (0.014)
TC pre-SMA	0.474 (0.047)	0.519 (0.027)
TC SMA	0.512 (0.030)	0.551 (0.002)
	*AD*	
TC Pre-SMA	0.566 (0.014)	0.580 (0.012)
TC SMA	0.492 (0.043)	0.559 (0.016)

*Numeric data are expressed in Spearman's rho (p-value). AD, axial diffusivity; EDSS, Expanded Disability Status Scale; IVF, isotropic volume fraction; NAWM, normal appearing white matter; SMA, supplementary motor area; TC, transcallosal*.

### Associations Between Brain/Lesion Volume and Clinical Measures of Disease

No significant associations were seen between whole brain and regional T1- and T2-lesion volume or BPF and the EDSS except for the significant associations found between the EDSS score and: (1) T1-lesion volume of the TC fibers of pre-supplementary motor area (*r* = 0.575, *p* = 0.016); (2) T1-lesion volume of the TC fibers of the dorsal premotor area (*r* = 0.521, *p* = 0.032); (3) T1-lesion volume of the TC fibers of the paracentral lobule (*r* = 0.637, *p* = 0.006); (4) T2-lesion volume of the TC fibers of the paracentral lobule (*r* = 0.628, *p* = 0.002); and (5) T1-lesion volume of the TC fibers of the primary motor area (*r* = 0.547, *p* = 0.023). On the contrary, the T25-FW was significantly associated with both the whole brain and each tract T2- and T1-lesion volumes although not with BPF. We depict these significant associations in Table 4.

**Table 4:** Significant correlations between tract-specific lesion volume and T25-FW.

	**T2-lesions**	**T1-lesions (black holes)**
*Whole brain*	0.525 (0.025)	0.620 (0.008)
TC Paracentral	0.621 (0.008)	0.506 (0.038)
TC-PMD	0.650 (0.005)	0.698 (0.002)
CS-PMD	0.553 (0.021)	0.661 (0.004)
TC-PMV	0.662 (0.004)	0.564 (0.018)
CS-PMV	0.586 (0.013)	0.484 (0.049)
TC-Pre-SMA	0.516 (0.034)	0.635 (0.006)
CS-Pre-SMA	0.623 (0.008)	0.654 (0.004)
TC-SMA	0.672 (0.003)	0.651 (0.005)
CS-SMA	0.664 (0.004)	0.734 (<0.001)
TC-M1	0.755 (<0.001)	0.725 (<0.001)
CS-M1	0.556 (0.020)	ns

*Numeric data are expressed in Spearman's rho (p-value). CS, corticospinal; ns: not significant; M1, primary motor cortex; PMD, dorsal premotor; PMV, ventral premotor; pre-SMA, pre-supplementary motor area; SMA, supplementary motor area; TC, transcallosal*.

## Discussion

In this proof-of-concept study, we tested the hypothesis if more pathologically- and topographically-specific measures of tissue injury improve our ability to link MRI with clinical metrics in pwMS.

Traditionally, the fiber assignment by continuous tractography (FACT) algorithm has been used to characterize WM tracts and quantify tract-specific metrics (40, 41). Leveraging FACT, fiber bundles are reconstructed via fiber trajectories generated from user-defined regions of interest (42, 43). Coupling the main limitation of FACT is its dependency on the DTI-derived fractional anisotropy metric, which is influenced by fiber crossing (43) and, in pwMS, the presence of lesions (10). Probabilistic tractography can overcome these limitations of FACT, by creating likelihood maps of connectivity in tracts of interest. However, interstudy differences, in how probabilistic tractography algorithms are conducted, could compromise generalizability of results.

To circumvent these limitations, researchers have sought to create high-resolution, freely available WM tractography templates. Leveraging tractography templates is a well-established method that has been used in a variety of neurodegenerative disorders, including stroke, Parkinsonism, and Alzheimer's disease (9, 20–22). There are several benefits to using these templates. First, these templates were developed using high-resolution (1.25 mm isotropic), multi-shell Human Connectome Project diffusion MRI data with high angular resolution (90+ direction acquisition). Second, tractography was conducted in 100 individuals and results were averaged to create a tractography template, which has minimal false positives/negatives. Coupling this approach with the NODDI, here we found that the IVF values of TC motor fiber bundles are higher in pwMS relative to HCs and in pwMS and motor disability relative to those without. The EDSS scores correlated significantly with the IVF values of the impaired tracts, while regional and whole-brain lesion burdens explained part of the variance of the T25-FW in a significant manner.

### IVF Differed Between pwMS and HCs as Well as Among pwMS With Various Degrees of Motor Impairment

The IVF differences were seen in TC projections from the paracentral lobule, the pre-supplementary and supplementary motor areas. These results held true when studying the WM of the entire tract, i.e., inclusive of lesions or the NAWM only. Similarly, removing individuals with secondary progressive MS did not change the findings.

The preponderant presence of subtle MS injury in TC tracts relative to CS ones is an interesting finding. Compared to CS fibers, TC fibers are in closer proximity to the ventricles, a factor that makes these bundles vulnerable to pro-inflammatory cerebrospinal fluid products and intrathecal immune activation. Previous literature supports this hypothesis. Combined MRI-positron emission tomography imaging studies using the mitochondrial translocator protein (TSPO) show enlarged and inflamed choroid plexuses in pwMS (44). Quantitative MRI studies show lower magnetization transfer ratio values and higher relative uptake of [^18^F]-DPA714 TSPO in areas located closer to the ventricles relative to voxels further away (45, 46). Our previous findings assessing differences between a total of 49 TC and CS tracts also showed that the latter ones tend to have larger lesion volumes, lower the ODI and NDI, and the higher IVF, highlighting once again the different venerability to disease between different groups of tracts (47).

When looking at differences in the specific NODDI metrics, our results only in part agree with those previously reported by others who also used a tract-based spatial statistics method to analyze differences in the NODDI between pwMS and HCs. Specifically, Hagiwara and collaborators (48) detected higher IVF values in several WM tracts in pwMS compared to HCs, but, contrary to our findings, they measured higher ODI values in the NAWM of the CS fibers originating from the primary motor cortex. The relatively small sample sizes of both he cohorts may be the factor skewing the results toward individual study cohort's demographics and the explanation for this inter-studies variability.

Increased IVF values in pwMS may represent a variety of biological alterations ranging from demyelination or neurodegeneration in WM fibers to edema yielding increased isotropic fluid diffusion (6). With respect to our cohort, we believe that lack of simultaneous differences in the NDI suggest that the IVF values likely represent early, e.g., prelesional, microstructural changes. These alterations are likely secondary to tissue disarrangement and extracellular water space enlargement and likely precede more severe and irreversible tissue injury, which we accordingly failed to identify on a group level. The robustness of our data was proven by similar findings generated with other microstructural models, e.g., AD from DTI and V_ax_ from the spherical mean technique.

### Associations Between the Neurite Orientation Dispersion and Density Imaging and Other MRI or Clinical Measures of Disease

The IVF values of the TC tracts of the paracentral lobule, pre-supplementary motor area, and supplementary motor area were higher in pwMS with motor disability relative to those without motor disability. Furthermore, disease in these TC tracts, as measured by the IVF values, was associated with the overall higher EDSS scores, but was not associated with focal lesion load. On the contrary, lesion load was significantly associated with the degree of impairment at the T25-FW.

Corticospinal tract injury typically produces weakness and spasticity; when motor neurons are involved as well, muscle atrophy develops due to denervation (49, 50). In pwMS, CS disease originates from both the brain and the spinal cord (16, 51). Thus, pathological changes of CS tracts are reflective of both the antegrade and retrograde injury propagation from either site. On the contrary, TC tracts connect homologous areas of the two hemispheres; thus, measurements of TC disease provide an estimate of only intracranial injury. TC fibers offer a higher level of regulation of motor functions through primarily net interhemispheric inhibitory effects (52–54). In MS, long-term injury of TC pathways may be viewed as an obstacle to functional recovery following rehabilitation strategies or acute intervention with steroids. Lack of recovery of motor function is certainly measurable by the motor score of the EDSS and can explain why differences in the IVF here seen between pwMS relative to HCs persisted when pwMS and motor disability were compared to those without. However, significant associations were here found between the IVF and the overall EDSS score. These data suggest that disease measured by the IVF in TC fibers was likely indicative of a more generalized diffuse process, represented by the overall EDSS score, than directly linked to motor impairment. In interpreting the data, one also must consider that the IVF is a metric with a relatively limited biological specificity relative to the NDI or ODI, a factor that may certainly account for our findings. In line with this statement, it is the similarity between the IVF and AD correlations output.

Accordingly, the T25-FW scores were significantly associated with regional volumes of lesions located in both the TC and CS tracts, but not with NAWM or the whole-tract IVF values. The findings confirm that subtle disease measured by the IVF is unlikely to directly impact function. More severe tissue injury, e.g., lesions, located in both the TC and CS tracts, is needed to affect walking ability and determine loss of function. It is noteworthy that associations were also seen between the EDSS and the regional lesion volume of several TC fiber bundles, indicating the ability of regional lesion volume to reflect a more broadly diffused disease pathology.

### Study Limitations and Conclusion

A few limitations of this study must be addressed to define an accurate framework of interpretation of our results. Specifically, this proof-of-concept cross-sectional study was based upon a small and an overall clinically heterogeneous pwMS cohort. These factors could have hidden group differences, generating some false-negative data and skewing the presented ones toward our demographics. Larger, longitudinal investigations are warranted to mitigate this weakness and to allow a more accurate generalizability of the data.

Furthermore, one may argue that none of the adopted templates has been previously used in a cohort of pwMS. However, besides the advantages of this approach discussed earlier in this section, we believe that it would be an inaccurate approach to conduct tractography in our specific cohort for several reasons: (1) this was a proof-of-concept study and, therefore, we have modest sample sizes, (2) there is a high variability in lesion location within our cohort, and (3) our diffusion MRI scans are not as high resolution as the HCP protocols. Given these reasons, the best alternative approach is to use existing tractography templates. An important next step in MS study is that of creating a large-scale cohort and compares various tractography methods, a goal that was beyond the scope of this manuscript.

Despite these important limitations, which form the basis for future study, this study confirmed the importance of TC along with CS fibers injury as a source of motor disability for pwMS.

## Data Availability Statement

The raw data supporting the conclusions of this article will be made available by the authors, without undue reservation.

## Ethics Statement

The studies involving human participants were reviewed and approved by Vanderbilt University Institutional Review Board. The patients/participants provided their written informed consent to participate in this study.

## Author Contributions

KY, DA, MC, SS, IO, GC, JX, and FB made substantial contributions to the conception or design of the study, the acquisition analysis or interpretation of data, or the creation of new software used in this study, drafted the study or revised it critically for important intellectual content, agree to be accountable for all the aspects of this study in ensuring that questions related to the accuracy or integrity of any part of this study are appropriately investigated and resolved. All authors contributed to the article and approved the submitted version.

## Funding

This study was supported by NIH/NINDS R01 NS094456 to IO, NMSS RD-1501-02840 to SS, NIH R01CA109106 to JX, the Clinical and Translational Science Award from National Cancer for Advanced Translational Sciences/NIH (grant UL1TR000445-06), NMSS RG-1901-33190, NINDS/NIH_R21 NS116434-01A1, and the Veterans Health Administration (1 I01 1 I01 CX002160-01) to FB.

## Author Disclaimer

These manuscript's contents are solely the responsibility of the authors and do not necessarily represent official views of the National Center for Advancing Translational Sciences or the National Institutes of Health (NIH).

## Conflict of Interest

The authors declare that the research was conducted in the absence of any commercial or financial relationships that could be construed as a potential conflict of interest.

## Publisher's Note

All claims expressed in this article are solely those of the authors and do not necessarily represent those of their affiliated organizations, or those of the publisher, the editors and the reviewers. Any product that may be evaluated in this article, or claim that may be made by its manufacturer, is not guaranteed or endorsed by the publisher.
