# Supplementary material for: Transcallosal and Corticospinal White Matter Disease and Its Association With Motor Impairment in Multiple Sclerosis
Source: Front Neurol. 2022 Jun 15;13:811315. doi: 10.3389/fneur.2022.811315 (PMC9240189; doi:10.3389/fneur.2022.811315)
Supplement: Supplementary file 1 [file Table_1.docx]

| Supplemental Table 1: Tract-specific lesion volume | | |
| --- | --- | --- |
|  | *T2-lesions (mm^3^)* | *T1-lesions (black holes, mm^3^)* |
| *TC Paracentral* | 109.7$\pm$167.7 | 43.3$\pm$64.7 |
| *TC-PMD* | 804.8$\pm$1453.7 | 320.1$\pm$319.8 |
| *CS-PMD* | 140.1$\pm$330.3 | 36.7$\pm$81.4 |
| *TC-PMV* | 514.9$\pm$849.6 | 273.8$\pm$267.0 |
| *CS-PMV* | 276.2$\pm$559.0 | 83.1$\pm$102.7 |
| *TC-Pre-SMA* | 320.6$\pm$552.8 | 100.0$\pm$147.2 |
| *CS-Pre-SMA* | 88.8$\pm$221.2 | 21.8$\pm$48.9 |
| *TC-SMA* | 391.4$\pm$715.9 | 144.6$\pm$152.1 |
| *CS-SMA* | 137.9$\pm$350.6 | 31.3$\pm$84.7 |
| *TC-M1* | 939.4$\pm$1495.8 | 373.2$\pm$429.7 |
| *CS-M1* | 717.5$\pm$1365.6 | 200.5$\pm$254.4 |
| Numeric data are expressed as mean$\boldsymbol{\pm}$standard deviation of the lesions volume in millimeters cubic. CS: corticospinal; mm^3^: cubic millimeters; M1: primary motor cortex; PMD: dorsal premotor; PMV: ventral premotor; pre-SMA: pre-supplementary motor area; SMA: supplementary motor area; TC: transcallosal. | | |
