# Supplementary material for: Transcallosal and Corticospinal White Matter Disease and Its Association With Motor Impairment in Multiple Sclerosis
Source: Front Neurol. 2022 Jun 15;13:811315. doi: 10.3389/fneur.2022.811315 (PMC9240189; doi:10.3389/fneur.2022.811315)
Supplement: Supplementary file 2 [file Table_2.docx]

| Supplemental Table 2: Tract-specific lesion volume in pwMS with and without motor disability | | | | |
| --- | --- | --- | --- | --- |
|  | *T2-lesions (mm^3^)* | | *T1-lesions (black holes, mm^3^)* | |
|  | pwMS with motor disability  (n=6) | pwMS without motor disability  (n=12) | pwMS with motor disability  (n=6) | pwMS without motor disability  (n=12) |
| *TC Paracentral* | 250.7$\pm$213.2 | 32.8$\pm$62.0 | 67.0$\pm$46.1 | 30.4$\pm$71.6 |
| *TC-PMD* | 1592.2$\pm$2259.2 | 375.3$\pm$504.4 | 574.2$\pm$255.9 | 181.5$\pm$266.5 |
| *CS-PMD* | 280.2$\pm$547.9 | 63.6$\pm$79.3 | 39.2$\pm$43.2 | 35.4$\pm$98.3 |
| *TC-PMV* | 1010.3$\pm$1283.0 | 244.7$\pm$322.8 | 470.8$\pm$183.5 | 166.4$\pm$247.6 |
| *CS-PMV* | 566.2$\pm$896.5 | 118.0$\pm$141.8 | 128.5$\pm$52.9 | 58.3$\pm$116.5 |
| *TC-Pre-SMA* | 627.3$\pm$832.0 | 153.4$\pm$235.6 | 211.5$\pm$197.4 | 39.2$\pm$60.6 |
| *CS-Pre-SMA* | 211.4$\pm$399.6 | 37.8$\pm$58.6 | 21.0$\pm$25.8 | 22.1$\pm$56.9 |
| *TC-SMA* | 826.0$\pm$1100.8 | 154.3$\pm$197.4 | 248.0$\pm$148.8 | 88.2$\pm$126.7 |
| *CS-SMA* | 314.0$\pm$570.3 | 41.9$\pm$72.4 | 28.7$\pm$41.5 | 32.7$\pm$103.0 |
| *TC-M1* | 1862.5$\pm$2260.6 | 435.9$\pm$484.4 | 544.0$\pm$297.3 | 280.1$\pm$473.6 |
| *CS-M1* | 1444.7$\pm$2151.3 | 320.8$\pm$423.4 | 269.3$\pm$173.2 | 162.9$\pm$290.1 |
| Numeric data are expressed as mean$\boldsymbol{\pm}$standard deviation of the lesions volume in millimeters cubic. CS: corticospinal; mm^3^: cubic millimeters; M1: primary motor cortex; PMD: dorsal premotor; PMV: ventral premotor; pre-SMA: pre-supplementary motor area; pwMS: people with multiple sclerosis; SMA: supplementary motor area; TC: transcallosal. | | | | |
