# Supplementary material for: Transcallosal and Corticospinal White Matter Disease and Its Association With Motor Impairment in Multiple Sclerosis
Source: Front Neurol. 2022 Jun 15;13:811315. doi: 10.3389/fneur.2022.811315 (PMC9240189; doi:10.3389/fneur.2022.811315)
Supplement: Supplementary file 3 [file Table_3.docx]

| Supplemental Table 3: Correlations between NAWM IVF / AD and lesion volume | | |
| --- | --- | --- |
|  | *T2-lesion volume* | *T1-lesion (black holes) volume* |
|  | *IVF* | |
| *TC Paracentral* | 0.290 (0.259) | 0.209 (0.420) |
| *TC Pre-SMA* | 0.172 (0.510) | 0.312 (0.223) |
| *TC SMA* | 0.252 (0.328) | 0.184 (0.479) |
|  | *AD* | |
| *TC Pre-SMA* | 0.350 (0.169) | 0.377 (0.135) |
| *TC SMA* | 0.403 (0.109) | 0.449 (0.071) |
| Numeric data are expressed in Spearman’s rho (p-value). AD: axial diffusivity; IVF: isotropic volume fraction; NAWM: normal appearing white matter; pre-SMA: pre-supplementary motor area; SMA: supplementary motor area; TC: transcallosal. | | |
